# A New Approach to the Degradation Stage Prediction of Rolling Bearings Using Hierarchical Grey Entropy and a Grey Bootstrap Markov Chain

**DOI:** 10.3390/s23229082

**Published:** 2023-11-09

**Authors:** Li Cheng, Wensuo Ma, Zuobin Gao

**Affiliations:** 1 School of Information Engineering, Henan University of Science and Technology, Luoyang 471023, China; 9945505@haust.edu.cn; 2 School of Mechatronics Engineering, Henan University of Science and Technology, Luoyang 471003, China; gaozuobin@haust.edu.cn

**Keywords:** rolling bearing, degradation stage prediction, hierarchical grey entropy, grey bootstrap Markov chain

## Abstract

Degradation stage prediction, which is crucial to monitoring the health condition of rolling bearings, can improve safety and reduce maintenance costs. In this paper, a novel degradation stage prediction method based on hierarchical grey entropy (HGE) and a grey bootstrap Markov chain (GBMC) is presented. Firstly, HGE is proposed as a new entropy that measures complexity, considers the degradation information embedded in both lower- and higher-frequency components and extracts the degradation features of rolling bearings. Then, the HGE values containing degradation information are fed to the prediction model, based on the GBMC, to obtain degradation stage prediction results more accurately. Meanwhile, three parameter indicators, namely the dynamic estimated interval, the reliability of the prediction result and dynamic uncertainty, are employed to evaluate the prediction results from different perspectives. The estimated interval reflects the upper and lower boundaries of the prediction results, the reliability reflects the credibility of the prediction results and the uncertainty reflects the dynamic fluctuation range of the prediction results. Finally, three rolling bearing run-to-failure experiments were conducted consecutively to validate the effectiveness of the proposed method, whose results indicate that HGE is superior to other entropies and the GBMC surpasses other existing rolling bearing degradation prediction methods; the prediction reliabilities are 90.91%, 90% and 83.87%, respectively.

## 1. Introduction

Rolling bearings are widely used in rotating machinery, and the performance degradation stage prediction of rolling bearings has attracted increasing attention [1,2,3]. The degradation forms of rolling bearings are diverse after experiencing long-term service, especially under harsh working and operating conditions. Such unexpected damage can make the mechanical system break down and result in enormous economic losses. Therefore, to maintain the safe and reliable operation of mechanical systems and keep a low downtime, it is of practical significance to predict the performance degradation stages of rolling bearings [4,5].

As their vibration signal characteristics are closely related to the physical structure of a rolling bearing, the vibration signal often contains abundant information about the bearing performance status and is sensitive to early weak failure and sudden failure [6,7]. Therefore, vibration-signal-based processing techniques are some of the current commonly used technical methods in rolling bearing degradation stage prediction. Degradation feature extraction is the key point that restricts the quality of prediction models, which require a strong dynamic response ability to describe the change in performance degradation degree [8]. However, the vibration signals of early faults in rolling bearings are very weak and are accompanied by strong background noise. In addition, the vibration signals of rolling bearings are nonlinear and non-stationary [9]. For this reason, traditional linear methods (e.g., time-domain-based analysis methods, frequency-domain-based analysis methods and time–frequency-domain-based analysis methods) cannot perform accurate extraction of rolling bearing degradation features. Therefore, developing a method that can maximize useful information without loss and perform the excellent extraction of degradation feature information hidden in vibration signals has been one of the critical challenges confronted by scholars in this field in recent years.

The entropy-based nonlinear dynamic method is one of the most powerful tools to detect the dynamic characteristics of time series [10,11,12]. Entropy is a material state parameter that reflects the irreversibility of spontaneous processes and is derived from the second law of thermodynamics. Shannon borrowed the concept of thermodynamics and called the average amount of information after eliminating redundancy in information “information entropy” [13], developing a mathematical expression to calculate information entropy. Kolmogorov and his student Sinai developed K-S entropy [14]. This research laid a solid theoretical foundation for the great progress of entropy theory.

In order to overcome the low computational efficiency of traditional nonlinear dynamic methods, Pincus proposed approximate entropy [15] (ApEn) to evaluate the complexity of a system. Sample entropy [16] (SE), proposed by Richman, is closer to the theoretical value than approximate entropy. Bandt proposed permutation entropy [17] (PE), which is more widely applicable. Costa introduced the concept of multiscale entropy [18] (ME) to address the complex temporal fluctuations inherent in physiological control systems that are not considered in single-scale analyses of health. However, the coarse-grained procedure used in ME essentially represents linear smoothing, which only captures low-frequency components using the averaging technique and ignores degradation information hidden in the high-frequency components [19]. Recently, Jiang et al. [20] put forward the concept of hierarchical entropy (HE) to evaluate the complexity of a time series, which proves to be an efficient method in considering the low-frequency and high-frequency components of time series. Compared with ME, the advantage of HE lies in the fact that it can reveal the inherent degradation characteristics of rolling bearing vibration signals in both low-frequency and high-frequency components by analyzing the complexity of different nodes, which indicates that it can be used to extract more comprehensive and accurate degradation feature information.

Due to the superiority of quantifying the uncertainty and dynamic change for a given time series, many entropy-based algorithms have been applied in the degradation feature extraction of rolling bearings. For instance, Wang et al. [21] proposed a degradation feature extraction technique based on equalization symbol sequence entropy. Song et al. [22] proposed a new nonlinear dynamic analysis method called refined composite multiscale amplitude-aware permutation entropy to extract features from bearing life data. Rai et al. [23] used multiscale fuzzy entropy as a fault feature that formed probability distributions, after which the Jensen–Renyi divergence technique was applied, which discriminates the probability distribution of degraded multiscale entropy feature vectors against healthy multiscale entropy feature vectors to formulate the desired health indicator. Li et al. [24] proposed a single-feature extraction method based on slope entropy and a double-feature extraction method based on slope entropy combined with permutation entropy. Chen et al. [25] proposed a novel method of fault feature extraction called hierarchical dispersion entropy.

In this paper, a new approach called hierarchical grey entropy (HGE) is first proposed to extract the degradation features of rolling bearings. HGE considers the degradation information embedded in both lower- and higher-frequency components. The lower- and higher-frequency components are generated using the moving-averaging procedure and moving-difference procedure in HGE, respectively. Therefore, HGE can provide a comprehensive evaluation of irregularity and uncertainty for a given time series.

In general, the process of rolling bearing degradation stage prediction contains three essential steps, data acquisition, degradation feature extraction and prediction model construction, and the latter two are the first priorities [26]. Therefore, after obtaining the degradation features, another important step is degradation stage prediction. Nowadays, machine learning is the most commonly used prediction model, which is because of its strong ability of nonlinear fitting [27,28]. A variety of machine learning approaches have been proposed to predict bearing performance. Gao et al. [29] proposed a prediction method of rolling bearing operational reliability based on isometric mapping and a nonhomogeneous cuckoo search–least squares support vector machine. Che et al. [30] proposed an intelligent fault prediction model based on gate recurrent units and hybrid autoencoders. Wu et al. [31] proposed a staged prediction method based on the regularized learning machine to predict remaining useful life of the bearing with high accuracy and speed. Xu et al. [32] proposed a remaining useful life prediction method of rolling bearing combining convolutional autoencoder networks and the status degradation model. Lan et al. [33] proposed a self-checking long- and short-term memory prediction model for predicting the performance degradation trend of bearings. Shang et al. [34] proposed an automated prediction technique based on a deep learning network for an end-to-end remaining useful life prediction of rolling bearings.

The research based on machine learning has achieved better results in the performance prediction of rolling bearings. However, machine learning methods are used under the condition that the sufficient samples are available, which is difficult to meet in degradation stage prediction of rolling bearings [35]. Grey prediction theories [36] have been proposed concerning specialties for a smaller number of sample attempts to explore development laws utilizing the mining of the internal regulation of data series, but these are still subject to lower prediction accuracy and higher time complexity. In this work, a new approach called the grey bootstrap Markov chain (GBMC) for rolling bearing degradation stage prediction is proposed through the combination of grey prediction theory, bootstrap method [37] and Markov chains [38]. The grey prediction model and the Markov chain are used to obtain the predicted value of the degradation stage, while the bootstrap method is used to establish a large sample prediction sequence of the degradation stage. Afterwards, the estimated true value is obtained from the statistical histogram of the large sample prediction sequence. At the same time, the dynamic estimation interval, prediction reliability and dynamic uncertainty are used to characterize the evolution trends of the performance degradation stage of rolling bearing.

To sum up, the novelties and contributions of this paper are as follows:

(1) HGE, overcoming the lack of high-frequency components in MGE analysis, is proposed to measure the complexity of time series. The experimental analysis of the noise signals and the rolling bearing vibration signals confirm that, compared to HSE and HFE, HGE has the advantage of lower data length requirement. Meanwhile, the degradation stage curves, obtained via HGE, are more consistent with real nonlinear dynamic systems.
(2) In view of the advantages of grey model, bootstrap method and Markov chain, the GBMC is presented for the degradation stage predictions of rolling bearings. Experimental analysis of the whole lifetime vibration data show that GBMC is superior to the GB model and AR method.
(3) Three parameter indicators are introduced to evaluate the prediction results from different perspectives. An experimental analysis shows that the estimated interval reflects the upper and lower boundaries of the prediction results, the reliability reflects the credibility of the prediction results and the uncertainty reflects the dynamic fluctuation range of the prediction results.

The rest of this article is organized as follows. The theory of HGE is developed in Section 2. Section 3 presents the theory of GBMC. Section 4 shows the flowchart of the proposed method for degradation stage prediction of rolling bearings. Section 5 validates the effectiveness and superiority of the proposed method through three experimental examples and contrastive analysis. In Section 6, conclusions are provided.

## 2. Hierarchical Grey Entropy

### 2.1. Grey Entropy

For a given time series of *N* points, {*y*_1_, *y*_2_, …, *y_t_*, …, *y_N_*}, *t* = 1, 2, …, *N*. The grey entropy (GE) can be defined as follows [39]:

Step 1. Reconstruct the time series into a series of phase orbits with embedding dimension *q* as expressed in Equation (1).

(1) $$\left[\begin{array}{cccc} y_{1} & y_{2} & \ldots & y_{G}\\ y_{2} & y_{3} & \ldots & y_{G+1}\\ \vdots & \vdots & \ldots & \vdots \\ y_{q} & y_{q+1} & \ldots & y_{G+q-1} \end{array}\right]$$

where *G* = *N* − *q* + 1.

Each column in the matrix can be viewed as a phase point vector *Y^q^*(*i*), which can be expressed as

(2) $$Y^{q}\left(i\right)=\left(y_{i},y_{i+1},\ldots ,y_{i+q-1}\right)$$

where *i* = 1, 2, …, *G*.

Step 2. Calculate the grey relational degree that indicates the similarity degree between the comparative sequences (*Y^q^*(*i*) and *Y^q^*(*j*)). The grey relational degree is defined as the following:

*Y^q^*(*i*) and *Y^q^*(*j*) are firstly normalized to obtain the new sequence as

(3) $$X^{q}\left(i\right)=\left(x_{1}^{i},x_{2}^{i},\ldots ,x_{k}^{i},\ldots ,x_{q}^{i}\right)$$

where *x*_1_*^i^*= *y_i_*/*y_i_* = 1, *x_q_^i^* = *y_i_*_+*q*−1_/*y_i_*, *k* = 1, 2, …, *q*.

(4) $$X^{q}\left(j\right)=\left(x_{1}^{j},x_{2}^{j},\ldots ,x_{k}^{j},\ldots ,x_{q}^{j}\right)$$

where *x*_1_*^j^*= *y_j_*/*y_j_* = 1, *x_q_^j^* = *y_j_*_+*q*−1_/*y_j_*.

Given the similarity tolerance *e*, *e* ∈ (0,1], the grey relational coefficient that indicates the closeness between *X^q^*(*i*) and *X^q^*(*j*) is defined as

(5) $$\delta_{k}^{ij}=\frac{\alpha_{min}+e\cdot \alpha_{max}}{\alpha_{k}^{ij}+e\cdot \alpha_{max}}$$

where

(6) $$\alpha_{k}^{ij}=\left|x_{k}^{i}-x_{k}^{j}\right|$$

(7) $$\alpha_{min}=min\left|x_{k}^{i}-x_{k}^{j}\right|$$

(8) $$\alpha_{max}=max\left|x_{k}^{i}-x_{k}^{j}\right|$$

We define the grey relational degree as

(9) $$\mu_{ij}^{q}\left(e\right)=\mu \left(X^{q}\left(i\right),X^{q}\left(j\right)\right)=\frac{1}{q}\sum_{k=1}^{q}\delta_{k}^{ij}$$

Step 3. The function *θ^q^*(*e*) is defined as

(10) $$\theta^{q}\left(e\right)=\frac{1}{N-q}\sum_{i=1}^{N-q}\left(\frac{1}{N-q}\sum_{j=1,j\neq i}^{N-q+1}\mu_{ij}^{q}\left(e\right)\right)$$

Similarly, the function *θ^q^*^+1^(*e*) for another given embedding dimension *q* + 1 can be defined as

(11) $$\theta^{q+1}\left(e\right)=\frac{1}{N-q}\sum_{i=1}^{N-q}\left(\frac{1}{N-q}\sum_{j=1,j\neq i}^{N-q+1}\mu_{ij}^{q+1}\left(e\right)\right)$$

Step 4. The GE can be calculated as expressed in Equation (12):

(12) $$GE\left(q,e\right)=\underset{N\to \infty }{\lim}\left(\ln\theta^{q}\left(e\right)-\ln\theta^{q+1}\left(e\right)\right)$$

The GE (*q*, *e*) of the original sequence is defined as the negative natural logarithm of the deviation of *θ^q^* from *θ^q^*^+1^. When the length *N* of time series is finite, GE (*q*, *e*) can be calculated as expressed in Equation (13):

(13) $$GE\left(q,e\right)=\ln\theta^{q}\left(e\right)-\ln\theta^{q+1}\left(e\right)$$

The physical meaning of *GE* is the negative natural logarithm of the conditional probability that two vectors similar for *q* points remain similar for the next *q* + 1 points, and the vectors’ similarity is determined by the grey relational degree. Physically, the smaller the GE, the lower the complexity of the time series, and the time series is treated as a low-complexity dynamical system that exhibits deterministic or periodic phenomena. The larger the GE, the higher the complexity of the time series, and the time series is treated as a high-complexity dynamical system that exhibits chaotic, stochastic or irregular phenomena.

### 2.2. Multiscale Grey Entropy

Based on the calculation processing of GE, multiscale grey entropy (MGE) can be calculated as follows.

MGE is defined as the GE of time series over different scales. For a discrete time series with length *N*: {*y_t_*, *t* = 1, 2, …, *N*}, given scale factor *λ*_max_, several coarse-grained time series can be constructed as

(14) $$Y^{\lambda }=\left(y_{1}^{\lambda },y_{2}^{\lambda },\ldots ,y_{i}^{\lambda },\ldots {,y}_{U}^{\lambda }\right)$$

where *λ* = 1, 2, …, *λ*_max_, *U* = *N*/*λ*, *i* = 1, 2, …, *U*, and

(15) $$y_{i}^{\lambda }=\frac{1}{\lambda }\sum_{t=\left(i-1\right)\lambda +1}^{i\lambda }y_{t}$$

In particular, when *λ* = 1, the coarse-grained series *Y^λ^* is simply the original time series {*y_t_*, *t* = 1, 2, …, *N*}.

We calculated GE for all coarse-grained time sequences *Y^λ^* with different scale factors one by one and then plotted these GE values as a function of scale factor *λ*. This procedure is called MGE analysis.

### 2.3. Hierarchical Grey Entropy

The MGE can effectively be used to extract the feature information hidden in the lower-frequency components for a given time series, but it ignores the feature information hidden in the high frequencies. To capture more useful feature information than the use of MGE, hierarchical grey entropy (HGE) is proposed in this paper. The proposed HGE can be calculated from the following steps:

Step 1. Define an averaging operator *T*_0_ for the time series *Y* = {*y_t_*, *t* = 1, 2, …, *N*} as

(16) $$T_{0}\left(Y\right)=\frac{y_{2i-1}+y_{2i}}{2},i=1,2,\ldots ,2^{m-1}$$

It should be noted that *N* = 2*^m^*, *m* is a positive integer, and *T*_0_(*Y*) with length 2*^m^*^−1^ is the low-frequency components of *Y* at scale 2. At the same time, another operator *T*_1_ is defined as

(17) $$T_{1}\left(Y\right)=\frac{y_{2i-1}-y_{2i}}{2},i=1,2,\ldots ,2^{m-1}$$

*T*_1_(*Y*) with length 2*^m^*^−1^ is the high-frequency components of *Y* at scale 2.

Step 2. For *j* = 0 or 1, the *f*-th layer operator $T_{j}^{f}$ is defined as

(18) $$T_{j}^{f}={\left[\begin{array}{ccccccc} \frac{1}{2} & \frac{{\left(-1\right)}^{j}}{2} & 0 & 0 & \ldots & 0 & 0\\ 0 & 0 & \frac{1}{2} & \frac{{\left(-1\right)}^{j}}{2} & \ldots & 0 & 0\\ \vdots & \vdots & \vdots & \vdots & \vdots & \vdots & \vdots \\ 0 & 0 & 0 & 0 & \ldots & \frac{1}{2} & \frac{{\left(-1\right)}^{j}}{2} \end{array}\right]}_{2^{m-f}\times 2^{m-f+1}}$$

Step 3. The hierarchical component *Y_f_*_,*g*_ for the *g*-th node in the *f*-th layer can be obtained by

(19) $$Y_{f,g}=T_{s_{f}}^{f}\cdot T_{s_{f-1}}^{f-1}\cdot \cdots \cdot T_{s_{1}}^{1}\cdot Y$$

where *f* is hierarchical layer number, *g* is hierarchical node number in the *f*-th layer and the one-dimensional vector {*s*_1_, *s*_2_, …, *s_f_*} ∈ {0, 1} can be calculated as expressed in Equation (20).

(20) $$g=\sum_{k=1}^{f}2^{f-k}s_{k}$$

Step 4. The GE of each hierarchical component *Y_f_*_,*g*_ is calculated to measure the complexity of the bearing vibration time series. This procedure is called HGE analysis.

**Figure 1:**
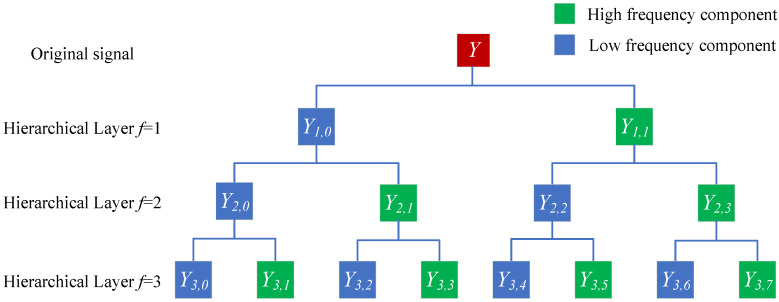
Hierarchical decomposition of *Y* with four scales.

The hierarchical decomposition of *Y* with four scales is given in Figure 1.

### 2.4. The Parameter Selection of HGE

**Figure 2:**
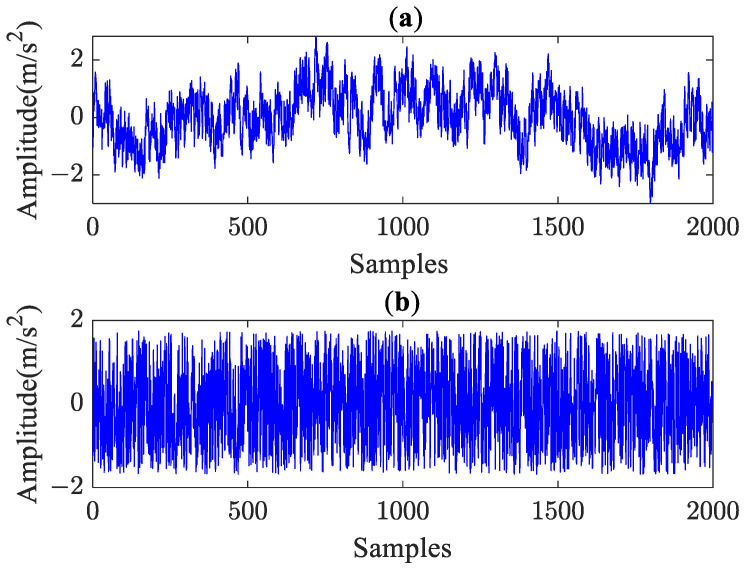
(**a**) Temporal waveform of 1/f noise. (**b**) Temporal waveform of Gaussian white noise.

In HGE analysis, three parameters need to be determined: the hierarchical layer number *f*, the embedding dimension *q*, and the similarity tolerance *e*. A synthetic noise signal of 1/f noise with data lengths *N* = 2000 was utilized for the test, and the temporal waveform of 1/f noise is plotted in Figure 2a. The specific steps of parameter selection are given as follows:

First, we investigated the hierarchical layer number of HGE analysis. Although a larger hierarchical layer could contain more lower- and higher-frequency information and enhance the classification accuracy, the computational complexity grows exponentially with the hierarchical layer [40]. In general, *f* depends on the length of experiment data and ranges from 1 to 3 [11,41]. In this paper, we set *f* = 2 with the hierarchical node of the final obtained decomposition features as 4.

**Figure 3:**
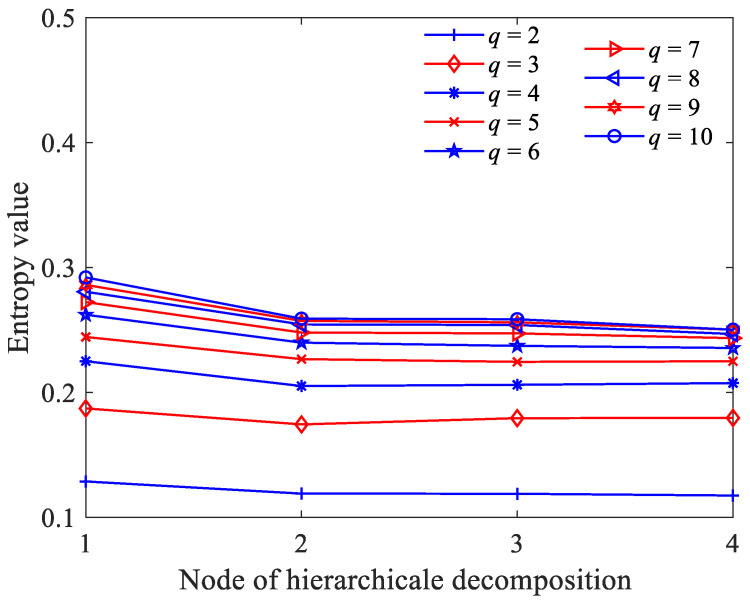
GE values of 1/f noise according to different embedding dimension *q* values.

Then, we explored the relationship between the GE values and embedding dimension *q*. Although the larger the *q* value, the richer the information obtained after phase space reconstruction, a too-large *q* value is disadvantageous owing to the need of a very large *N* (10*^q^*–30*^q^*), which is difficult to meet for a bearing vibration time series [22]. Figure 3 illustrates the GE values of 1/f noise according to different embedding dimension *q* values; we found that with the increase in *q* value, GE also gradually increases and converges to about 0.3. It is worth noting that when *q* ≥ 3, the fluctuation trend at different nodes no longer changes. This indicates that the GE with embedding dimension *q* = 3 is sufficient to accurately reflect the dynamic changes. Therefore, the embedding dimension *q* was set as 3.

**Figure 4:**
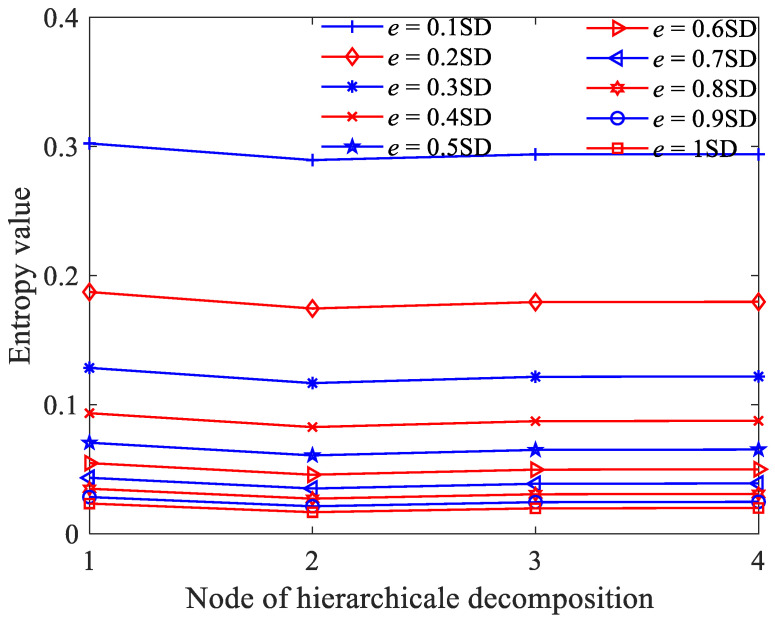
GE values of 1/f noise according to different similarity tolerance *e* values.

At last, we considered the selection of the similarity tolerance *e*. A larger similarity tolerance *e* could lead to more difficult matching of templates and much more statistical information being lost, while a small similarity tolerance *e* could make the evaluated result of statistical properties inaccurate and sensitive to noise. Figure 4 shows the obtained results using different similarity tolerance *e* values. From Figure 4, it can be found that with the increase in *e* value, GE gradually decreases and converges to about 0.02. When *q* ≤ 0.2 SD (SD is the standard deviation of the original signal), the fluctuation trend at different nodes no longer changes. This indicates that the GE with similarity tolerance *e* = 0.2 SD is sufficient to accurately reflect the dynamic changes. Therefore, the similarity tolerance *e* was set as 0.2 SD.

**Figure 5:**
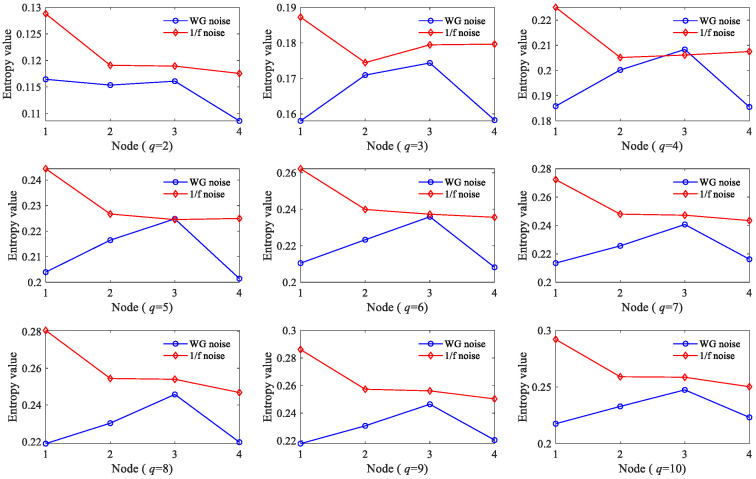
HGE with different embedding dimension *q* values.

**Figure 6:**
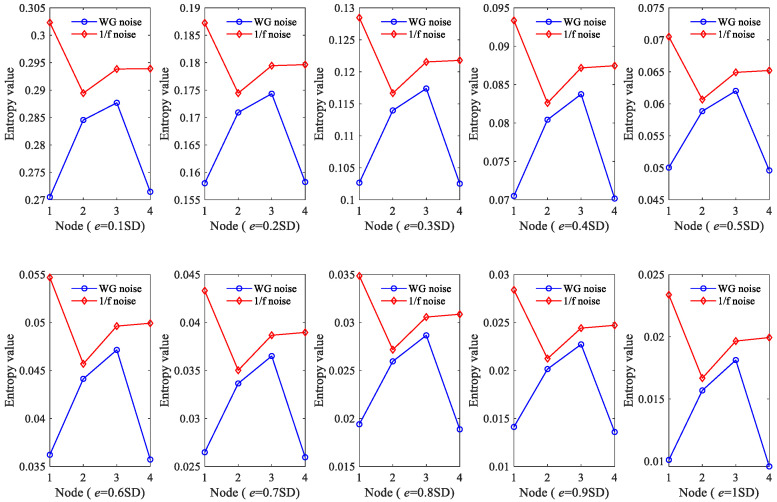
HGE with different similarity tolerance *e* values.

In order to perform a more in-depth discussion on how these parameters affect the performance of HGE, the GE values obtained for 1/f noise and white gaussian noise (WG noise) with different *q* values (2, 3, 4, 5, 6, 7, 8, 9 and 10) and *e* values (0.1 SD, 0.2 SD, 0.3 SD, 0.4 SD, 0.5 SD, 0.6 SD, 0.7 SD, 0.8 SD, 0.9 SD and 1 SD) were calculated successively. For fair comparison, in Figure 5, the parameters were set as *e* = 0.2 SD, *f* = 2 and in Figure 6, the parameters were set as *q* = 3, *f* = 2, separately. The results are shown in Figure 5 and Figure 6 separately. It can be found clearly from Figure 5 that HGE can separate two signals when *q* = 2, *q* = 3, *q* = 7, *q* = 8, *q* = 9 and *q* = 10. Considering that if *q* is too large, the value of data length *N* is required to be large, resulting in complicated computation, it is reasonable to choose *q* = 3. From Figure 6, it can be seen that for all *e* values, HGE can be used to separate the two signals. This shows that the selection of *e* hardly affects the performance of HGE, that is, HGE has good robustness for the selection of *e*.

### 2.5. Comparison Analysis

To verify the superiority of the proposed method, 1/f noise and WG noise containing 2000 data points were used to implement the comparisons between HGE, the hierarchical fuzzy entropy (HFE) and the hierarchical sample entropy (HSE) methods. Figure 2 shows the randomly selected temporal waveforms of 1/f noise and WG noise, respectively. It can be seen from Figure 2 that the fluctuation trend of WG is relatively stable; in contrast, 1/f noise has a more complex structure and contains more mode information. Therefore, 1/f noise is more complex than WG noise regarding structure.

**Figure 7:**
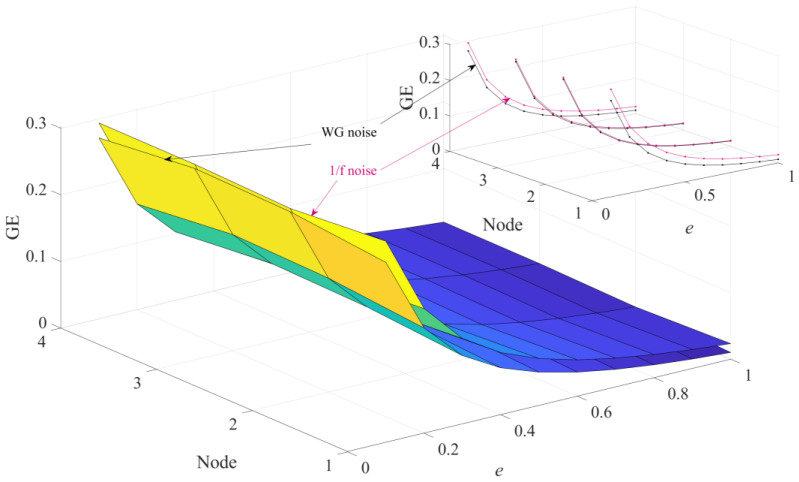
HGE analysis of 1/f noise and WG noise.

**Figure 8:**
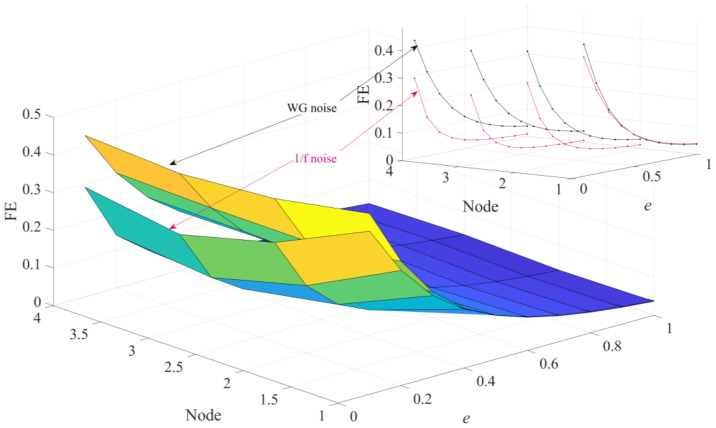
HFE analysis of 1/f noise and WG noise.

**Figure 9:**
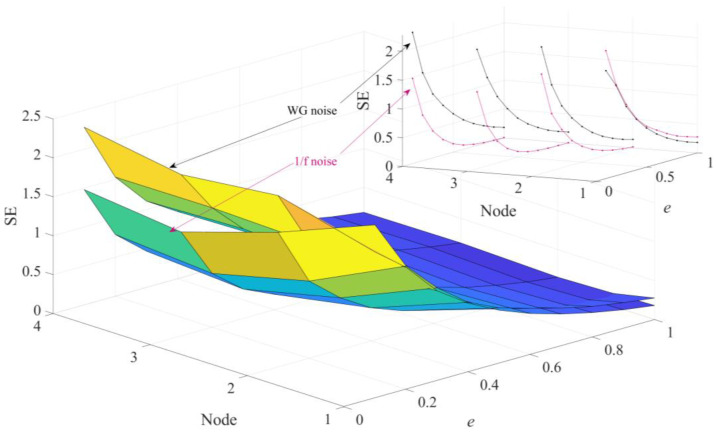
HSE analysis of 1/f noise and WG noise.

To explore the estimation performance of HGE, HFE and HSE methods, the HGE, HFE and HSE were employed to analyze the WG noise and 1/f noises across four nodes. Figure 7, Figure 8 and Figure 9 show the performance of HSE, HFE and HGE on these signals respectively, where embedding dimension *q* = 3. As shown in Figure 7, it can be found that the GE of both 1/f noise and WG noise obtained by HGE decreases monotonically with the increase in similarity tolerance *e*, and the GE values of 1/f noise are all greater than those of WG noise in the four nodes, which is in accordance with the theoretical analysis. This illustrates that the HGE can separate WG noise and 1/f noise well.

Seen from Figure 8, although HFE can separate WG noise and 1/f noise in the last three nodes (node = 2, 3, 4), the FE values of WG noise are greater than those of 1/f noise, which is not consistent with the theoretical analysis. From Figure 9, it can be clearly found that though HSE shows good relative consistency when the node is 1, it no longer holds the property when the value of node is greater than 1.

Therefore, compared with HFE and HSE, HGE can extract entire information and effectively describe dynamic changes of the complex signal.

## 3. Grey Bootstrap Markov Chain

The grey bootstrap Markov chain (GBMC) model is a combination model based on the grey model, bootstrap method and Markov chain. The modeling process of GBMC is described as follows.

### 3.1. Markov Chain Theory

Assuming the stochastic process {*c_n_*, *n* ∈ *T*} for any states {*i*_0_, *i*_1_, …, *i_n_*} = *I* and integers *T* = {0, 1, …} satisfies

(21) $$P\left\{c_{n+1}=i_{n+1}|c_{0}=i_{0},c_{1}=i_{1},\cdots ,c_{n}=i_{n}\right\}\;\;\;\;\;\;=P\left\{c_{n+1}=i_{n+1}|c_{n}=i_{n}\right\}$$

Then the stochastic process {*c_n_*, *n* ∈ *T*} is said to have the Markovian property. A stochastic process is a Markov chain if it has the Markovian property.

Suppose a set of data {*c*(*t*), *t* ∈ *T*} is a Markov chain, the state space is {*I*_1_, *I*_2_, …, *I_m_*} and each state has *m* transitions. Taking *I*_1_ as an example, the possible transitions can be *I*_1_→*I*_1_, *I*_1_→*I*_2_, …, *I*_1_→*I_m_*.

The *k*-step transition probability *p*^(*k*)^*_ij_* is the conditional probability that the system will be in state *I_j_* after *k* steps, given that it starts in state *I_i_*, which can be calculated as follows:

(22) $${p^{\left(k\right)}}_{ij}=P\left(I_{i}\to I_{j}\right)={N^{\left(k\right)}}_{ij}/N_{i}$$

where *N_i_* is the number of states *I_i_* and *N*^(*k*)^*_ij_* is the number of states *I_i_* that can be transited to state *I_j_*. 

Thus, the *k*-step transition probability matrix can be expressed as:

(23) $$P^{\left(k\right)}=\left[\begin{array}{cccc} {p^{\left(k\right)}}_{11} & {p^{\left(k\right)}}_{12} & \ldots & {p^{\left(k\right)}}_{1n}\\ {p^{\left(k\right)}}_{21} & {p^{\left(k\right)}}_{22} & \ldots & {p^{\left(k\right)}}_{2n}\\ \vdots & \vdots & \vdots & \vdots \\ {p^{\left(k\right)}}_{n1} & {p^{\left(k\right)}}_{n2} & \ldots & {p^{\left(k\right)}}_{nn} \end{array}\right]$$

The *p*^(*k*)^*_ij_* must satisfy the properties

(24) $${p^{\left(k\right)}}_{ij}\geq 0,\;i,j=1,2,\ldots ,n$$

and

(25) $$\sum_{j=1}^{n}{p^{\left(k\right)}}_{ij}=1,\;i=1,2,\ldots ,n$$

### 3.2. Grey Bootstrap Markov Chain Method

For a given time series of *N* points, *R* = {*r* (1), *r* (2), …, *r* (*t*), …, *r* (*M*)}, *t* = 1, 2, …, *M*, taking *v* data adjacent to time *t* from *R* (including the data at time *t*) to form the rolling fusion subsequence of time *t* as follows:

(26) $$R_{v}=\left\{r_{v}\left(u\right),u=t-v+1,t-v+2,\cdots ,t\right\},\;t\geq v$$

Based on *R_v_*, the bootstrap method was used to obtain the resampling samples *Q*_bootstrap_ as follows:

(27) $$Q_{bootstrap}=\left[Q_{1},Q_{2},...,Q_{b},...,Q_{B}\right]$$

where *Q_b_* is the *b*-th bootstrap sample and *Q_b_* = {*q_b_*(*i*)}, *i* = 1, 2, …, *v*, *b* = 1, 2, …, *B*, *q_b_*(*i*) represents the *i*-th bootstrap resampling data in *Q_b_*.

The predicted value ${\hat{q}}_{b}\left(w\right)$ of *Q_b_* at time *w* = *t* + 1 is obtained via the GM (1,1) model [42], and the predicted sequence generated by accumulation is:

(28) $${\hat{Q}}_{b}=\left\{{\hat{q}}_{b}(i)\right\},\;,i=1,2,\cdots ,v$$

The residual between the original sequence and the predicted sequence is

(29) $$\varepsilon_{b}\left(i\right)=q_{b}(i)-{\hat{q}}_{b}(i)$$

Let

(30) $${\bar{\varepsilon }}_{b}(i)=\left(\left|q_{b}(i)-{\hat{q}}_{b}(i)\right|\right)$$

We define the Markov interval span as

(31) $$D_{b}=\frac{\varepsilon_{b,max}-\varepsilon_{b,min}}{m}$$

where *m* is the number of Markov state intervals, and

(32) $$\varepsilon_{b,max}=\max\left|q_{b}(i)-{\hat{q}}_{b}(i)\right|,\;\varepsilon_{b,min}=\min\left|q_{b}(i)-{\hat{q}}_{b}(i)\right|$$

We divided *D_b_* into *m* states; *I_b_*, *j* represents the *j*-th state, *I_b_*_,*j*_ = [*ρ_b_*_,*j*−1_, *ρ_b_*_,*j*_], *j* = 1,2,…,*m*. The more state intervals are divided, the higher the prediction accuracy. However, if there are too many state intervals, the number of samples in each interval is too small, which actually affects the prediction accuracy. Therefore, appropriate state divisions should be made according to the actual situation. For this article, we divided the interval into four groups, which can be expressed as

(33) $$I_{b,j}=\left[\varepsilon_{b,min}+D_{b}\left(j-1\right),\varepsilon_{b,min}+D_{b}j\right],\;j=1,2,3,4$$

We conducted the Markov states partition of ${\bar{\varepsilon }}_{b}$ and constructed a *k*-step transition probability matrix as follows:

(34) $$P_{b}^{\left(k\right)}=\left[\begin{array}{cccc} p_{11}^{\left(k\right)} & p_{12}^{\left(k\right)} & \cdots & p_{1m}^{\left(k\right)}\\ p_{21}^{\left(k\right)} & p_{22}^{\left(k\right)} & \cdots & p_{2m}^{\left(k\right)}\\ \vdots & \vdots & \vdots & \vdots \\ p_{m1}^{\left(k\right)} & p_{m2}^{\left(k\right)} & \cdots & p_{mm}^{\left(k\right)} \end{array}\right]$$

We selected the *m* residuals in the residual sequence *ε_b_* closest to time *w* and then selected their corresponding row vector in the *k*-step transition probability matrix to form the *m*-order state transition probability matrix as follows:

(35) $${\hat{P}}_{b}=\left[\begin{array}{cccc} {\hat{p}}_{i1}^{\left(1\right)} & {\hat{p}}_{i2}^{\left(1\right)} & \cdots & {\hat{p}}_{im}^{\left(1\right)}\\ {\hat{p}}_{i1}^{\left(2\right)} & {\hat{p}}_{i2}^{\left(2\right)} & \cdots & {\hat{p}}_{im}^{\left(2\right)}\\ \vdots & \vdots & \vdots & \vdots \\ {\hat{p}}_{i1}^{\left(m\right)} & {\hat{p}}_{i2}^{\left(m\right)} & \cdots & {\hat{p}}_{im}^{\left(m\right)} \end{array}\right]$$

where *i* is the Markov state of the residual.

The probability of *m*-order Markov chains can be calculated as follows:

(36) $${\left.P\right|}_{I_{b,j}}=\sum_{k=1}^{m}{\hat{p}}_{ij}^{\left(k\right)},\;j=1,2,\ldots ,m$$

We took the state *I_b_*_,*j*_ corresponding to $\max\left\{{P|}_{I_{b,j}},I_{b,j}\in I_{b}\right\}$ as the next best state predicted by the bootstrap Markov chain, and then the predicted residual value at time *w* was obtained as:

(37) $$\varepsilon_{b}\left(w\right)=\frac{\rho_{b,j-1}+\rho_{b,j}}{2}$$

Let state 1 indicate a positive residual sign and state 2 indicate a negative residual sign. Conduct the Markov symbol states partition of $\varepsilon_{b}$, and a one-step transition probability matrix can be obtained as follows:

(38) $$P_{b}=\left[\begin{array}{cc} p_{11} & p_{12}\\ p_{21} & p_{22} \end{array}\right]$$

where *p_ij_* = *N_ij_*/*N_i_*, *i*, *j* = 1,2, *N_i_* is the number of states *i*, and *N_ij_* is the number of states *i* that can be transited to state *j*.

Define initial state *p*^(0)^ = [0.5, 0.5], and then the probability of residual sign state can be calculated as follows:

(39) $$P_{b}^{\left(w\right)}=p^{\left(0\right)}P_{b}=\left[p_{b,1}^{\left(w\right)},p_{b,2}^{\left(w\right)}\right]$$

The predicted residual sign at time *w* can be obtained as:

(40) $$f_{b}\left(w\right)=\left\{\begin{array}{cc} 1, & p_{b,1}^{\left(w\right)}-p_{b,2}^{\left(w\right)}\geq 0\\ -1, & p_{b,1}^{\left(w\right)}-p_{b,2}^{\left(w\right)}<0 \end{array}\right.$$

The GM (1,1) prediction value revised by the bootstrap Markov chain is

(41) $$q_{b}^{*}\left(w\right)={\hat{q}}_{b}\left(w\right)+f_{b}\left(w\right)\varepsilon_{b}\left(w\right)$$

### 3.3. Parameter Indicators for Dynamic Evaluation

At time *w* = *t* + 1, there are *B* prediction values, which can form the sequence as follows:

(42) $$r_{w}^{*}=\left\{q_{b}^{*}\left(w\right)\right\},b=1,2,\cdots ,B$$

Afterwards, the frequency function of *r_v_* at time *t* can be established based on $r_{w}^{*}$ as follows:

(43) $$F_{w}=F_{w}\left(r_{v}\right)$$

Draw a statistical histogram of $r_{w}^{*}$ based on statistical principles, and the median value *ζ_z_* and frequency *c_z_* of each group can be obtained.

(1) Estimated true value

At time *w*, the estimated true value can be expressed as:

(44) $$X_{0}\left(w\right)=\frac{1}{Z}\sum_{z=1}^{Z}c_{z}\zeta_{z}$$

(2) Dynamic estimated interval

Let the significance level be *σ*; then the confidence level can be obtained as

(45) H=(1−σ)×100%

At time *w*, when the confidence level is *H*, the estimated interval for the true value of the attribute parameter is [*X_L_*, *X_U_*]; *X_L_* and *X_U_* can be calculated respectively as follows:

(46) $$X_{L}=\sigma \left(K_{U}-K_{L}\right)+K_{L}$$

(47) $$X_{U}=\left(1-\sigma \right)\left(K_{U}-K_{L}\right)+K_{L}$$

where *K_L_* is the lower limit value of the first group of histograms, and *K_U_* is the upper limit value of the *z*-th group of histograms.

(3) Reliability of prediction result

For a sequence with length *T*, there are *h* attribute parameter values outside the estimation interval [*X_L_*, *X_U_*], and the reliability of the grey bootstrap Markov chain prediction model can be defined as:

(48) $$H_{B}=\left(1-\frac{h}{T-v+1}\right)\times 100\%$$

(4) Dynamic uncertainty

Define the dynamic uncertainty of the attribute parameters at time w as

(49) $$U=U(w)=X_{U}-X_{L}$$

## 4. The Degradation Stage Prediction Procedure

**Figure 10:**
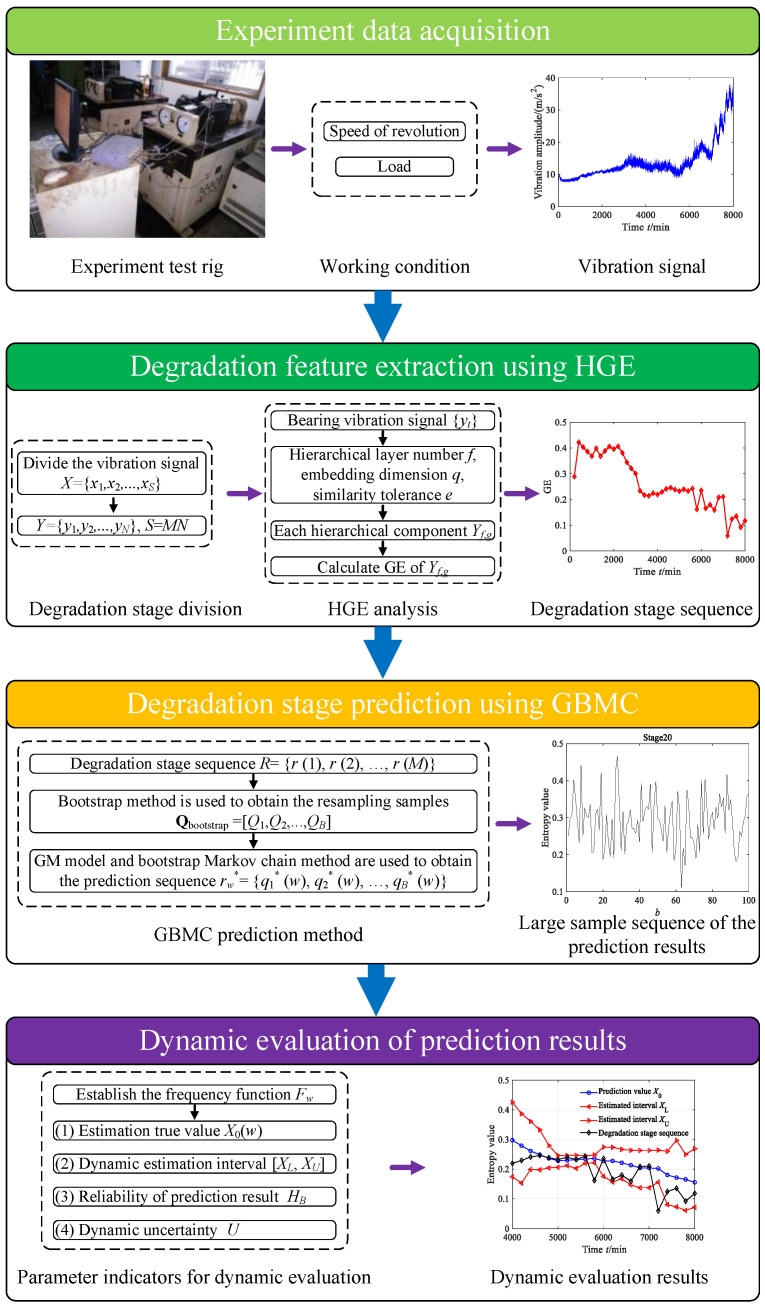
Flowchart of the proposed degradation stage prediction method.

Based on HGE and GBMC, a novel degradation stage prediction method for rolling bearings is proposed in this paper. Figure 10 shows the flowchart of the proposed scheme, and its main processes are described as follows:

Step 1. Conduct fatigue life testing and collect the vibrational signals under different working conditions.

Step 2. Employ the HGE employed to extract the degradation stage feature from the collected vibrational signals. Firstly, divide the vibration signal into *M* degradation stages. Then, perform HGE analysis on each degradation stage, and take the GE value of each stage as the degradation feature value, where *q* = 3, *e* = 0.2 SD, *f* = 2, node = 1. Finally, combine the degradation feature values of each stage to form a degradation stage sequence.

Step 3. Employ the proposed GBMC method to predict the degradation stage. Firstly, use the bootstrap method to obtain the large sample sequence of the degradation stage. Then, use the grey Markov chain method to make a one-step prediction of the large sample sequence of the degradation stage. Finally, use the prediction results to establish the large sample prediction sequence of the degradation stage.

Step 4. Apply three parameter indicators to evaluate the prediction results. Firstly, establish a frequency function based on the large sample prediction sequence of the degradation stage. Then, obtain the estimated true values, that is, the predicted values, for each degradation stage using the frequency function. Finally, employ three parameter indicators, namely the dynamic estimated interval, reliability of prediction result and dynamic uncertainty, to evaluate the prediction results from different perspectives.

## 5. Experiment Verification

In order to validate the effectiveness of the proposed method in this paper for rolling bearing degradation stage prediction, three experimental cases were conducted consecutively.

### 5.1. Case 1

**Figure 11:**
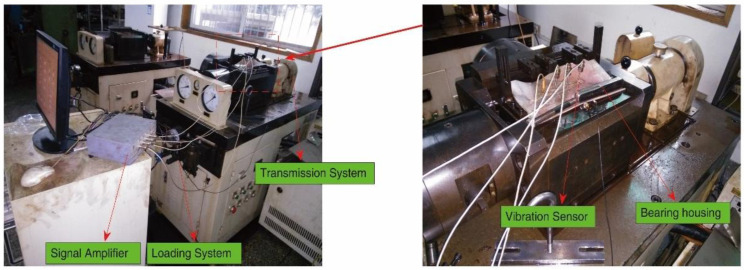
The test rig.

The accelerated life test was implemented at the Hangzhou Bearing Test and Research Center to acquire the whole-lifetime vibration data. The test rig consisted of a monitoring system, transmission system, loading system, lubrication system and computer control system as shown in Figure 11. The bearing life enhancement test machine was ABLT-1A from Hangzhou Bearing Test and Research Center Co., Ltd., Hangzhou, China, the acceleration sensor was AI002 from Yangzhou Jingming Technology Co., Ltd., Yangzhou, China, and the dynamic signal test and analysis system was JM5937 from Yangzhou Jingming Technology Co., Ltd., Yangzhou, China. The bearings used in the test were tapered roller bearings 7008 AC from Luoyang Bearing Research Institute Co., Ltd., Luoyang, China. Table 1 and Table 2 show the test conditions and bearing parameters, respectively.

One datum was recorded every minute. In order to observe the complete degradation process of the bearing from normal state to minor failure and then to severe failure, the relative method was used in the experiment to determine the failure threshold of the bearing. When the maximum amplitude of the bearing vibration signal exceeded 3A (A is the maximum amplitude of the bearing during normal operation), the bearing was considered to have completely failed and the test was immediately terminated. The accelerated life test was carried out successively until the crest factor of vibration signal exceeded the set value; the total number of sampling vibration data was 8000.

**Figure 12:**
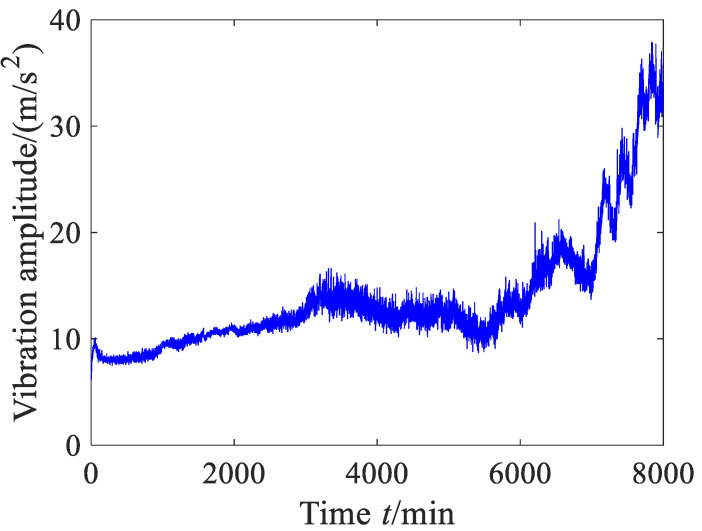
Time domain wave of the vibration signal (case 1).

The vibration data in this case are exactly the same as those of Experiment 2 in Reference [39]. The waveform of the original vibration signal is shown in Figure 12, and the vibration data did not undergo any preprocessing. From Figure 12, it can be found that the vibration amplitude has an upward trend. Before 200 min, the vibration amplitude slightly decreases as time goes on and then enters a stable stage, indicating that the rolling bearing enters the normal operation period after a short run-in period. From around 3000 min, the vibration amplitude increases and then begins to decrease, being basically in a fluctuating state. At this stage, the rolling bearing may have undergone slight failure and started to enter the failure period. After 6000 min, the vibration amplitude sharply increases in fluctuation. At this stage, the rolling bearing already experienced serious failure.

**Figure 13:**
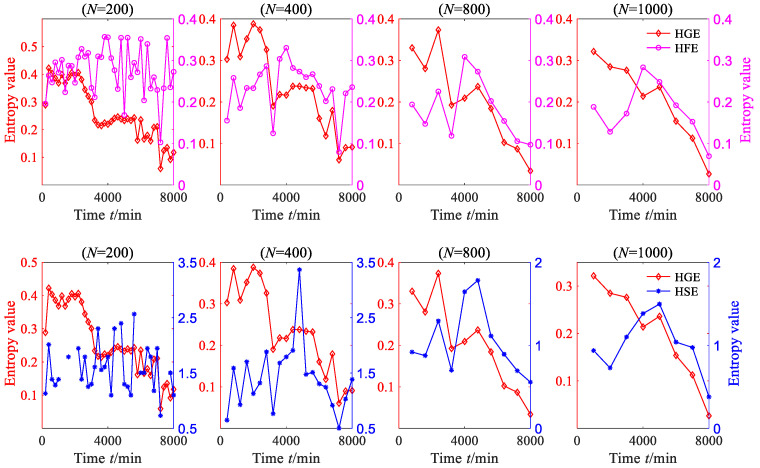
The HGE, HFE and HSE of rolling bearing vibration signal (case 1).

A sequence with *N* data is used to represent a degradation stage, so the vibration signal can be divided into 8000/*N* degradation stages. The HGE of each degradation stage was calculated to obtain the degradation feature sequence of rolling bearing. In order to investigate the effect of data length *N* on HGE, HFE and HSE calculations, these three statistics were all applied to analyze degradation stages with different lengths—*N* = 200, *N* = 400, *N* = 800 and *N* = 1000—and the results are shown in Figure 13, where *q* = 3, *e* = 0.2 SD, *f* = 2, node = 1. It can be seen from Figure 13 that for four different lengths of degradation stages, the degradation feature sequences, which were extracted using HGE entropy, show a phased downward trend overall.

According to reference [16], the entropy algorithm measures the complexity and regularity of nonlinear signals. When the rolling bearing is in normal operation, its motion is extremely irregular, which means that the complexity of the vibration signal of the rolling bearing is the highest. When local fault defects occur, the vibration signal of the rolling bearing exhibits obvious periodic signal characteristics, which means that the complexity of the vibration signal is reduced. When the defects of rolling bearings further expand, the periodic characteristics of their vibration signals becomes more obvious, which means that the complexity of the vibration signals is further reduced. The results calculated via HGE are consistent with the above phenomenon, which indicates that the results calculated via the proposed method are consistent with real nonlinear dynamic systems. It is worth mentioning that when the degradation stage length *N* = 200, HGE characterizes the degradation trend of rolling bearings more accurately, which indicates that HGE has a lower requirement for data length and that reliable calculation results can be obtained with a shorter data length.

To demonstrate the superiority of HGE, the HSE and HFE of the degradation stage were calculated separately under the same parameters, as shown in Figure 13. From Figure 13, it can be seen that when *N* = 200 and *N* = 400, the degraded feature sequences based on HSE and HFE do not exhibit obvious regularity; when *N* = 800 and *N* = 1000, the degraded feature sequences based on HSE and HFE show a decreasing trend only when *t* > 4000 min.

**Figure 14:**
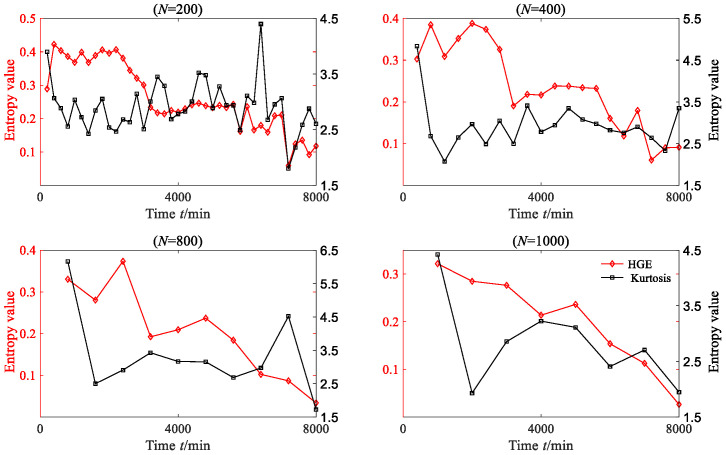
The HGE and kurtosis of rolling bearing vibration signal (case 1).

In order to convincingly establish the superiority of HGE, kurtosis, which is a commonly used time-domain feature extraction method, was employed to extract the rolling bearing degraded features. The kurtosis values of each degradation stage were calculated, as shown in Figure 14. From Figure 14, it can be found that when *N* = 200, *N* = 400 and *N* = 800, the degraded feature sequences based on kurtosis do not exhibit obvious regularity. Although the degradation feature sequence based on kurtosis shows a downward trend when *N* = 1000, this trend is not consistent with the evolution trend of vibration amplitude.

The above analysis shows that HGE presents an inherently more effective measure for characterizing the degradation feature of rolling bearings than HSE, HFE and kurtosis, and HGE also has the advantage of lower data length requirements. Therefore, HGE can be used to extract the degradation feature of rolling bearings.

The large time span caused by calculating the HGE of each degradation stage when the sample length is too long is not conducive to the timely mining of degradation information during the operation of rolling bearings. In this article, we set 200 data as a degradation stage (*N* = 200), and the vibration signal was divided into 40 degradation stages. The HGE of each degradation stage was calculated to obtain the degradation feature sequence of rolling bearings.

**Figure 15:**
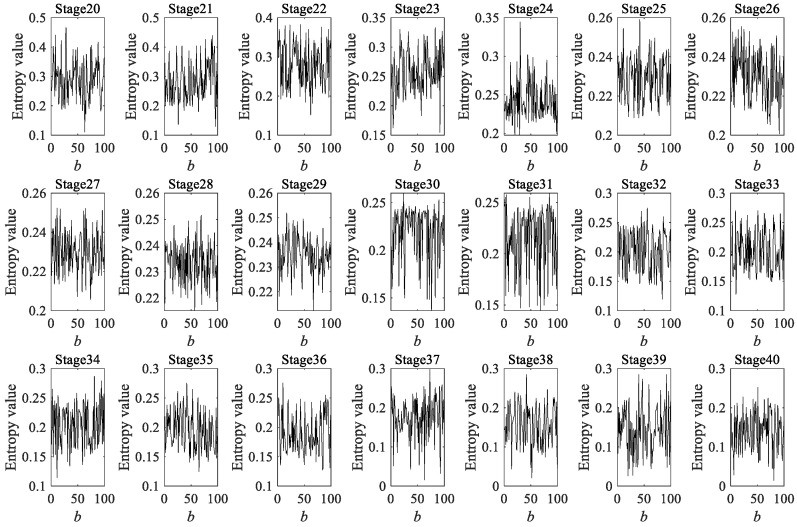
Large sample sequence of the degradation stages (case 1).

**Figure 16:**
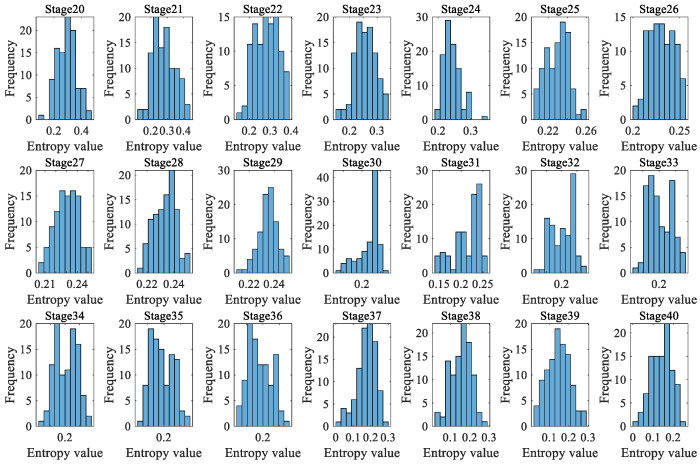
Histogram of large sample sequences of degradation stages (case 1).

Due to the fact that a rolling bearing is in the optimal operation period for the first 4000 min, there is no practical significance to predicting its degradation during this stage. Therefore, a dynamic prediction was made for the degradation stage sequence corresponding to the last 4000 min (stage 20 to 40, a total of 21 degradation stages). The large sample sequence of 21 degradation stages predicted via the GBMC model is shown in Figure 15. We sorted the data in the large sample sequence of 21 degradation stages from small to large and then grouped them, taking *Z* = 10. We drew a histogram, shown in Figure 16.

**Figure 17:**
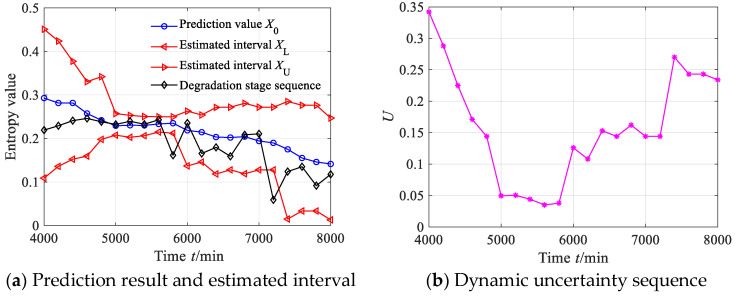
Prediction results and dynamic evaluation of rolling bearing degradation stages based on GBMC model (case 1).

According to Equation (44), we calculated the estimated true values of 21 degradation stages to obtain the degradation feature prediction sequence. According to Equations (45)–(47), the estimated interval of the degradation stage sequence can be obtained where the significance level is set as 0.05, as shown in Figure 17a. From Figure 17a, it can be seen that the prediction sequence of the degradation stage generally shows a downward trend. Between 4000 and 6000 min, the prediction sequence of the degradation stage is almost consistent with the degradation stage sequence. Between 6000 and 8000 min, due to the continuous degradation of the bearing vibration performance, the extracted degradation stage values show a disorderly fluctuation state, but the prediction sequence still captures the continuous downward trend of the degradation stage. From Figure 17a, it can also be found that the estimated interval of the degradation stage sequence almost includes all the degradation stage values, with only two degradation stage values falling outside the estimated interval. According to Equation (48), the prediction reliability *H_B_* is 90.91%, indicating that the estimated interval can effectively track the evolution trend of the degradation stage sequence.

The dynamic uncertainty of the degradation stage sequence can be obtained according to Equation (49), as shown in Figure 17b. From Figure 17b, it is clear that the uncertainty shows a decreasing trend between 4000 and 5000 min, indicating that after slight degradation of the rolling bearing, it begins to enter a “self-healing” period and the uncertainty begins to decrease. Between 5000 and 5800 min, the uncertainty shows a stable fluctuation trend, indicating that the rolling bearing enters a brief period of stable operation after “self-healing”, and the uncertainty stabilizes at a small value stage; after 5800 min, the uncertainty shows a continuous upward trend, indicating further deterioration of the rolling bearing.

From the above analysis, it is clear that the evolution process of the degradation stage of rolling bearings is characterized from different perspectives by the estimated true value *X*_0_ (prediction value), estimated interval [*X_L_*, *X_U_*], prediction reliability *H_B_*, and dynamic uncertainty *U*. The estimated true value reflects the evolution trend of the degradation stage of rolling bearings, the estimated interval reflects the upper and lower boundaries of the prediction results, the reliability reflects the credibility of the prediction results and the uncertainty reflects the dynamic fluctuation range of the prediction results.

**Figure 18:**
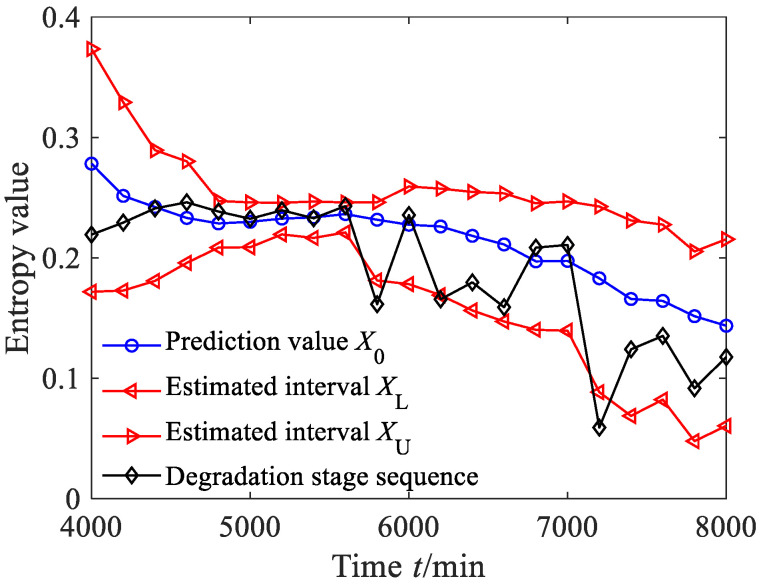
Prediction results of rolling bearing degradation stages based on GB model (case 1).

In order to verify the superiority of the proposed model, the grey bootstrap (GB) model [43], which combines grey prediction theory and the bootstrap method, was used to predict the degradation stages, as shown in Figure 18. From Figure 18, it can be seen that although the prediction value of the degradation stage obtained via the GB model shows an overall downward trend, which partly reflects the evolution trend of the degradation stage, there are more true values of the degradation stage that fall outside the estimated interval than GBMC, and the prediction reliability *H_B_* is only 86.36%, indicating that the estimated interval cannot effectively capture the range of the rolling bearing degradation stage.

**Figure 19:**
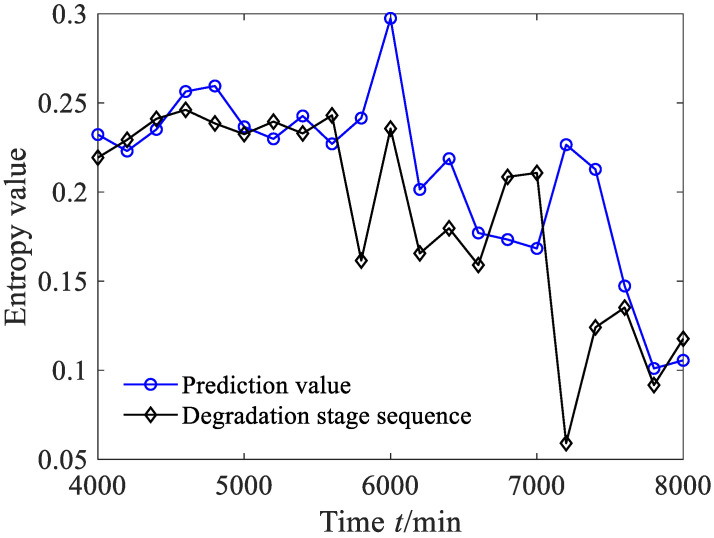
Prediction results of rolling bearing degradation stages based on AR method (case 1).

For comparison, the AR method was also used to predict the degradation stage of the bearing, and the results are shown in Figure 19. The performance of GBMC and AR was evaluated based on average absolute error and correlation coefficient, and the comparison results are listed in Table 3. Consulting Table 3, although the prediction errors of bearing degradation stages obtained via the AR method are slightly smaller than the GBMC, the correlation coefficient obtained via the GBMC is larger than that obtained via the AR method. It should also be pointed out that compared to the AR method, the GBMC has the advantage of dynamic evaluation of prediction results.

Through the above analysis, it is clear that compared with the GB model and AR method, the proposed GBMC method of this paper can effectively predict and evaluate the degradation stage of rolling bearing. This provides a new prediction and evaluation method for the online monitoring and operational performance evaluation of rolling bearings.

### 5.2. Case 2

**Figure 20:**
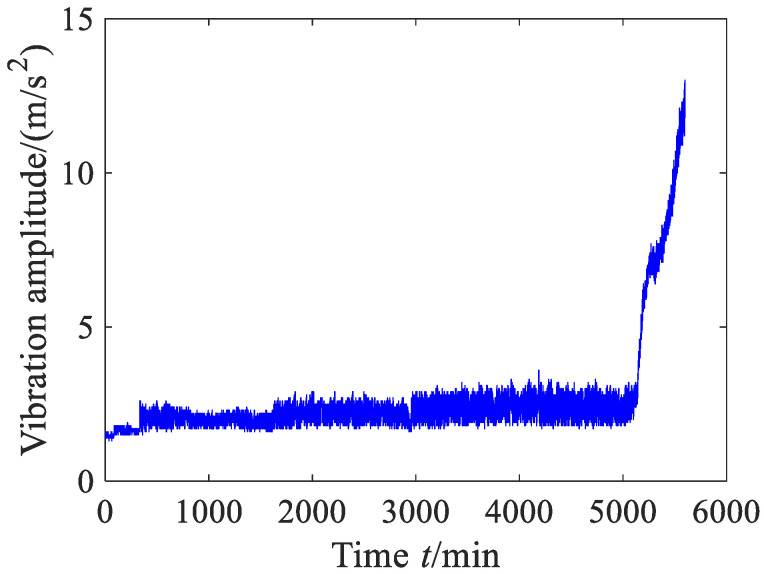
Time domain wave of the vibration signal (case 2).

To illustrate the universality of the proposed method, another accelerated life test was also conducted at the Hangzhou Bearing Test and Research Center, where the basic layout of the test rig was the same as in case 1. Table 4 lists the detailed test conditions. The total number of sampling vibration data is 5600. The vibration data in this case are exactly the same as those of Experiment 3 in Reference [39]. The vibration data did not undergo any preprocessing. The waveform of the vibration signal is shown in Figure 20. From Figure 20, it can be seen that the vibration amplitude of the bearing remains at a small value between 0 min and 400 min, indicating that the bearing was in the initial running in period; during 401–5000 min, the vibration amplitude of the bearing is in a stable fluctuation state, indicating that the bearing was in a normal operating period; and after 5000 min, the vibration amplitude of the bearing rapidly increases, indicating that the bearing entered a deterioration period.

**Figure 21:**
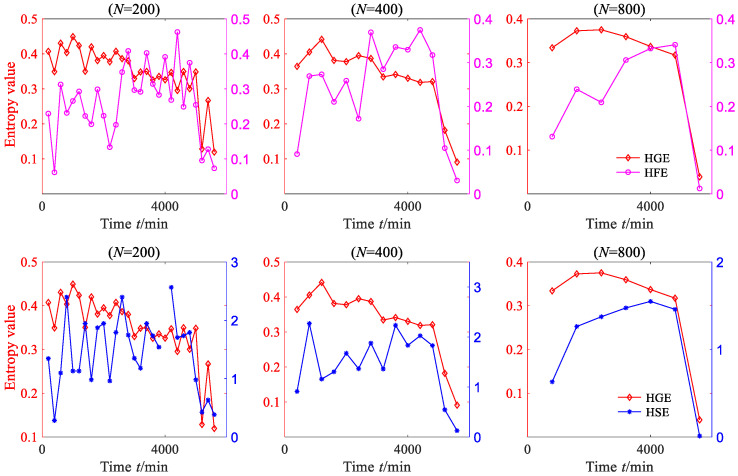
The HGE, HFE and HSE of rolling bearing vibration signal (case 2).

**Figure 22:**
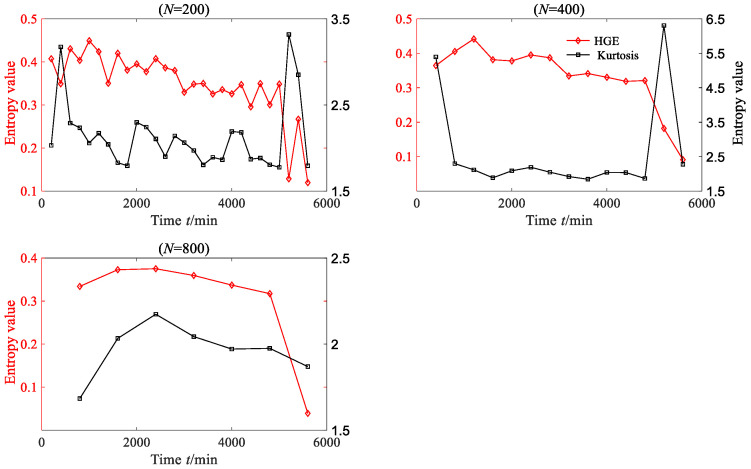
The HGE and kurtosis of rolling bearing vibration signal (case 2).

HGE, HFE and HSE were all applied to analyze degradation stage with three different lengths: *N* = 200, *N* = 400, *N* = 800, where *q* = 3, *e* = 0.2 SD, *f* = 2, node = 3; the results are shown in Figure 21. From Figure 21, it is clear that for three different data lengths, the degradation feature sequences extracted based on HGE can accurately depict the degradation trend of bearing. In contrast, the degradation features extracted based on HFE and HSE cannot effectively reflect the degradation trend of bearings, especially when the data length of each degradation stage is short (*N* = 200); the extracted degradation feature sequence shows an irregular state. The kurtosis values of each degradation stage were calculated, as shown in Figure 22. From Figure 22, it can be seen that for these three data lengths, the degraded feature sequences based on kurtosis did not exhibit significant regularity.

**Figure 23:**
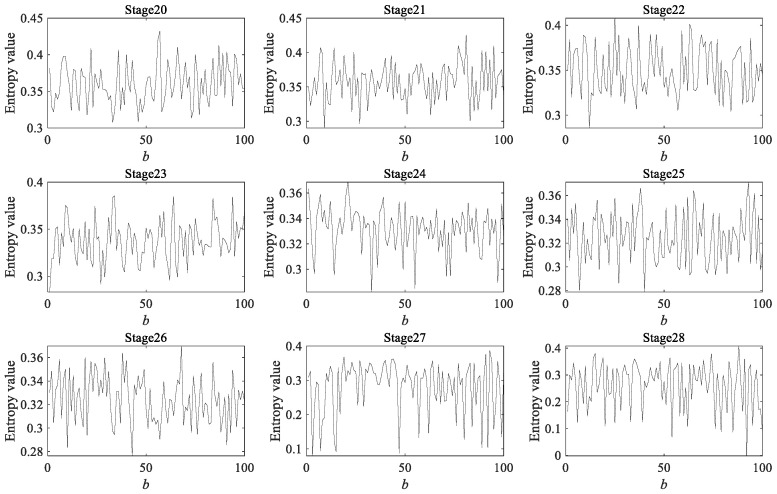
Large sample sequence of the degradation stages (case 2).

**Figure 24:**
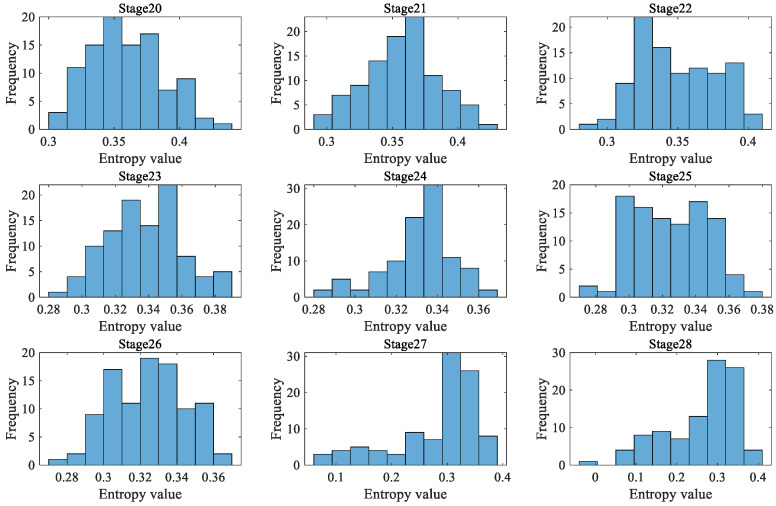
Histogram of large sample sequences of degradation stages (case 2).

As in case 1, 200 data were taken as a degradation stage (*N* = 200), and the vibration signal was divided into 28 degradation stages. We calculated the HGE of each degradation stage to obtain the degradation feature sequence of the rolling bearings. The dynamic prediction was made for the degradation stage sequence corresponding to the last 1600 min (stages 20 to 28, a total of 9 degradation stages). The large sample sequence of nine degradation stages predicted by the GBMC model is shown in Figure 23. We sorted the data in the large sample sequence of nine degradation stages from small to large, and then grouped them, taking *Z* = 10. We drew a histogram, shown in Figure 24.

**Figure 25:**
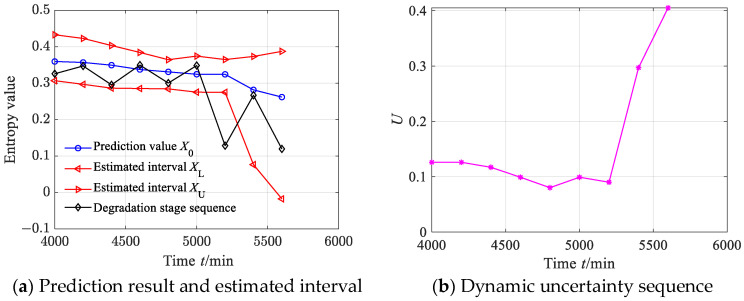
Prediction results and dynamic evaluation of rolling bearing degradation stages based on GBMC model (case 2).

According to Equation (44), we calculated the estimated true values of nine degradation stages to obtain the degradation feature prediction sequence. According to Equations (45)–(47), the estimated interval of the degradation stage sequence was obtained, where the significance level was set as 0.05, as shown in Figure 25a. From Figure 25a, it can be seen that between 4000 and 5000 min, the prediction value of the degradation stage is almost consistent with the degradation stage value. Between 5000 and 5600 min, the predicted sequence captured the downward trend of the degradation stage. From Figure 25a, it can also be found that the estimated interval almost includes all the degradation feature values, with only one degradation feature value falling outside the estimated interval. According to Equation (48), the prediction reliability *H_B_* is 90%, indicating that the estimated interval can effectively track the evolution trend of the degradation stage. The dynamic uncertainty of the degradation stage sequence was calculated as shown in Figure 25b. From Figure 25b, it is clear that between 4000 and 5000 min, the uncertainty fluctuates steadily, indicating that the bearing was in a stable operating period and the uncertainty stabilized at a small value stage; after 5000 min, the uncertainty rapidly increased, indicating that the rolling bearing underwent serious deterioration.

**Figure 26:**
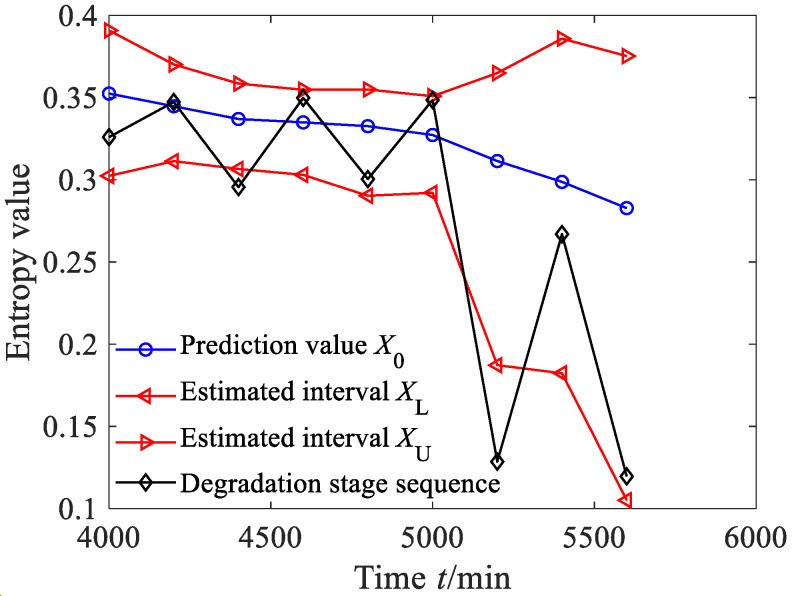
Prediction results of rolling bearing degradation stages based on GB model (case 2).

**Figure 27:**
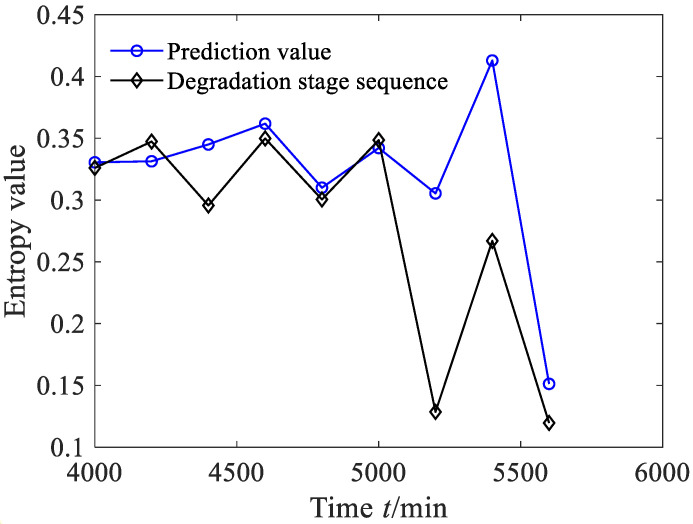
Prediction results of rolling bearing degradation stages based on AR method (case 2).

The prediction results based on the GB model are shown in Figure 26, from which it can be found that although the prediction value of the degradation stage obtained via the GB model shows an overall downward trend, reflecting to some extent the evolution trend of the rolling bearing degradation stage; the prediction reliability *H_B_* is only 80%. The prediction results based on the AR method are shown in Figure 27, and the comparison results are listed in Table 5. To see Table 5, although the prediction errors and the correlation coefficient of the bearing degradation stages obtained via the AR method are almost the same as that of the GBMC; the GBMC has the advantage of dynamic evaluation of prediction results.

Therefore, the above analysis indicates that the proposed GBMC-based degradation stage prediction method is superior to the GB-based and AR-based degradation stage prediction methods.

### 5.3. Case 3

**Figure 28:**
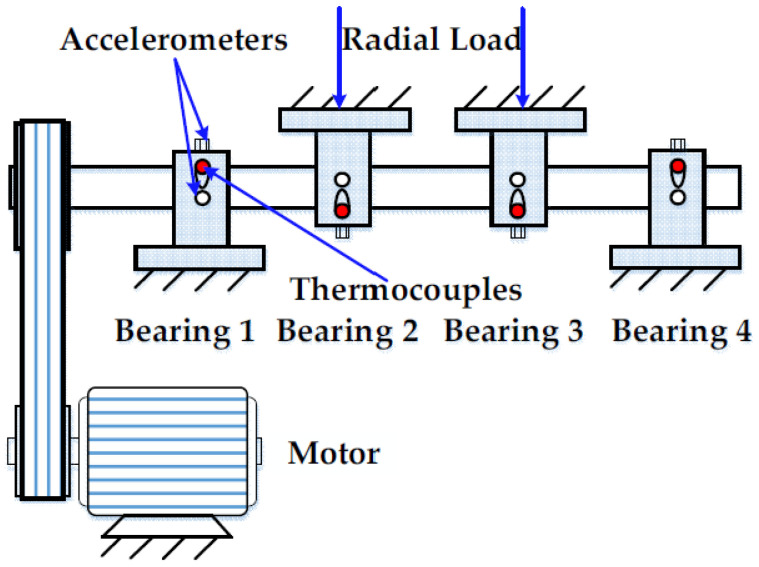
Schematic of the bearing test rig.

**Figure 29:**
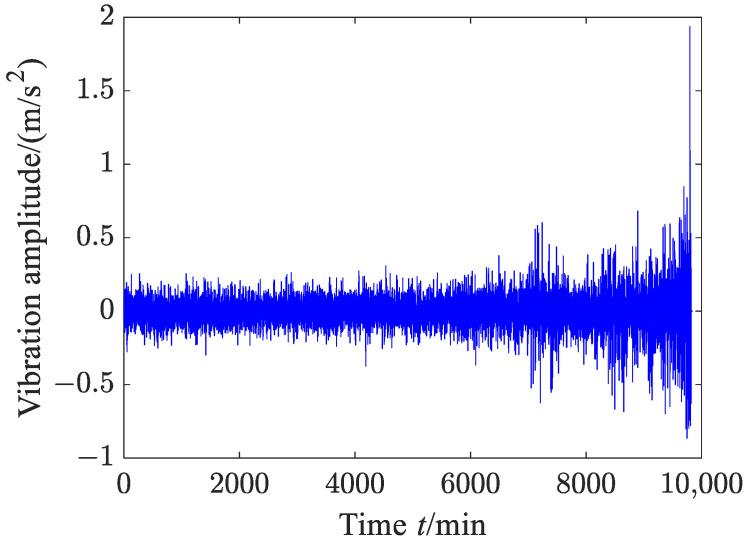
Time domain wave of the vibration signal (case 3).

The experimental data were the full life cycle vibration data of rolling bearings from the Center for Intelligent Maintenance Systems (IMS), University of Cincinnati [44]. The test rig [11] consisted of a spindle, four test bearings, an AC motor and a rub belt, as shown in Figure 28. The test bearing was a Rexnord ZA-2115 double row bearing. The shaft was driven by an AC motor and coupled by rub belts. A radial load of 6000 lb was added to the shaft and bearing by a spring mechanism. The rotation speed was 2000 rpm. The accelerometer was installed on each bearing housing. The sampling frequency was 20 kHz, and each file contained 20 480 data. The collection interval between each two files was 10 min. The data collection started at 10:32:39 on 12 February 2004 and ended at 6:22:39 on 19 February 2004. A total of 984 files were collected, and the experiment lasted for 163 8 h. At the end, the outer ring of bearing 1 was seriously deteriorated. Due to the large amount of data in each package and the fluctuation range of magnitude being almost consistent, 10 representative data were selected from each package to form the bearing life data, the data length was 984 × 10 and the waveform of the vibration data is shown in Figure 29.

It can be seen from Figure 29 that the vibration amplitude of the rolling bearing is relatively stable before 7000 min and the fluctuation range is not large, indicating that the rolling bearing is in the normal operation period. From 7000 min, the vibration amplitude of rolling bearings begins to gradually increase, indicating that rolling bearing performance begins to deteriorate until it fails.

**Figure 30:**
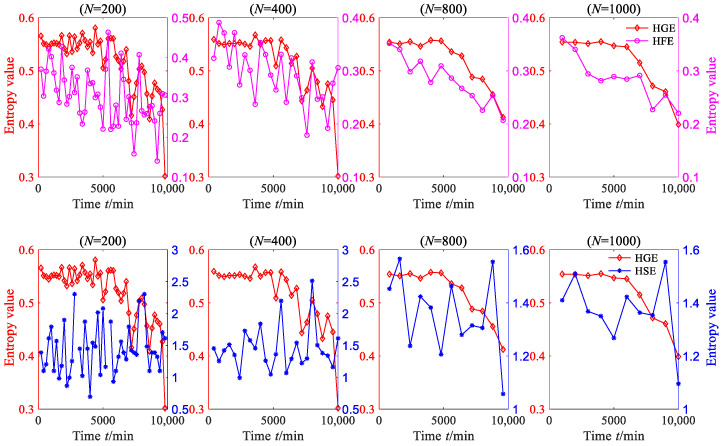
The HGE, HFE and HSE of rolling bearing vibration signal (case 3).

**Figure 31:**
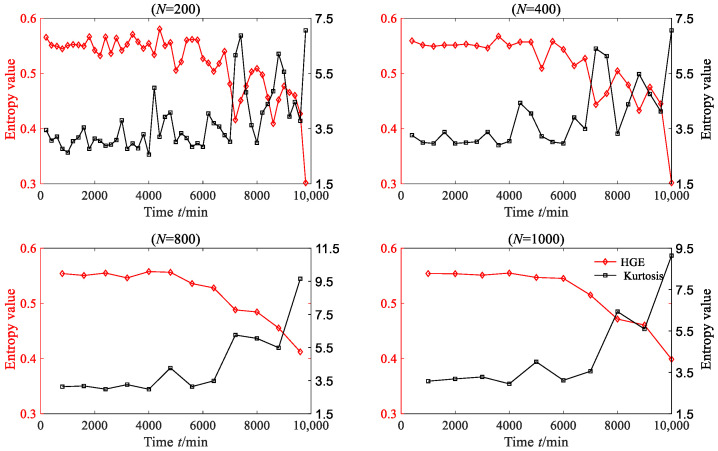
The HGE and kurtosis of rolling bearing vibration signal (case 3).

HGE, HFE and HSE were all applied to analyze degradation stage with three different lengths: *N* = 200, *N* = 400, *N* = 800 and *N* = 1000, where *q* = 3, *e* = 0.2 SD, *f* = 2, node = 3; the results are shown in Figure 30. From Figure 30, it is clear that for four different data lengths, the degradation feature sequences extracted based on HGE can accurately depict the degradation trend of the bearing. Before 7000 min, the entropy value of HGE fluctuates smoothly with little change, while after 7000 min, the entropy value of HGE begins to gradually decline. The trend in HGE is consistent with the trend in rolling bearing vibration signal. In contrast, the degradation features extracted based on HFE and HSE cannot effectively reflect the degradation trend of bearings, especially when the data length of each degradation stage is short (*N* = 200 and *N* = 400); the extracted degradation feature sequence shows an irregular state. The kurtosis values of each degradation stage were calculated, as shown in Figure 31. From Figure 31, it can be found that when *N* = 200 and *N* = 400, the degraded feature sequences based on kurtosis do not exhibit obvious regularity. Although the degradation feature sequence based on kurtosis shows an upward trend when *N* = 800 and *N* = 1000, this trend is opposite to the degradation trend of rolling bearings.

**Figure 32:**
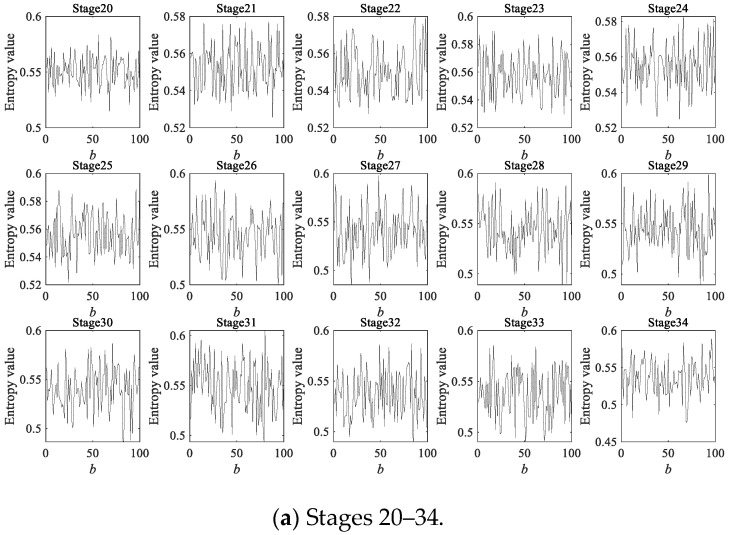

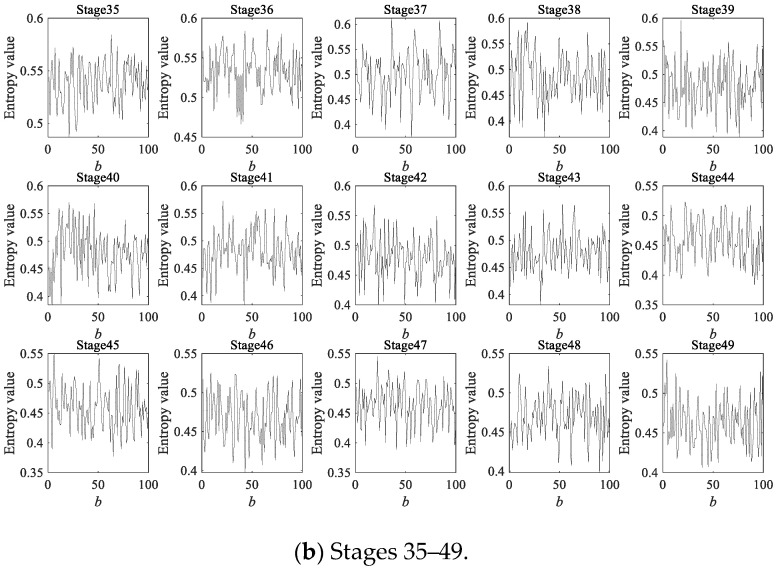
Large sample sequence of the degradation stages (case 3).

**Figure 33:**
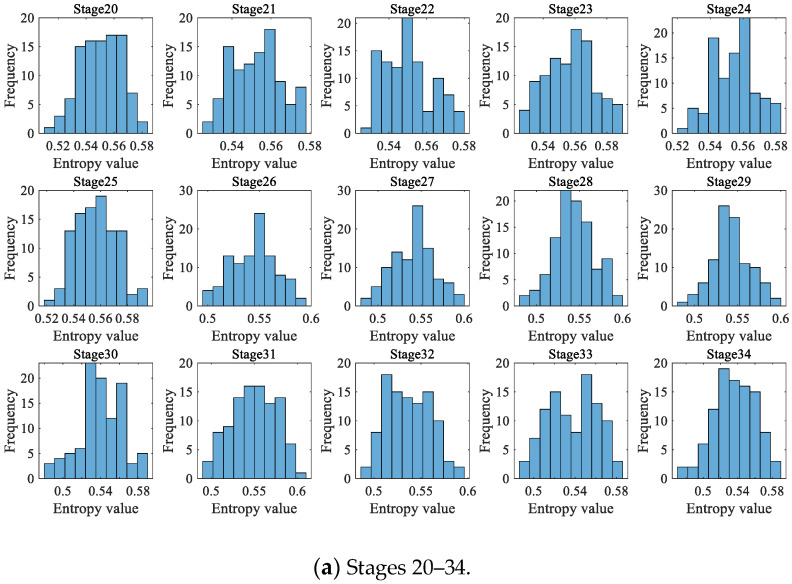

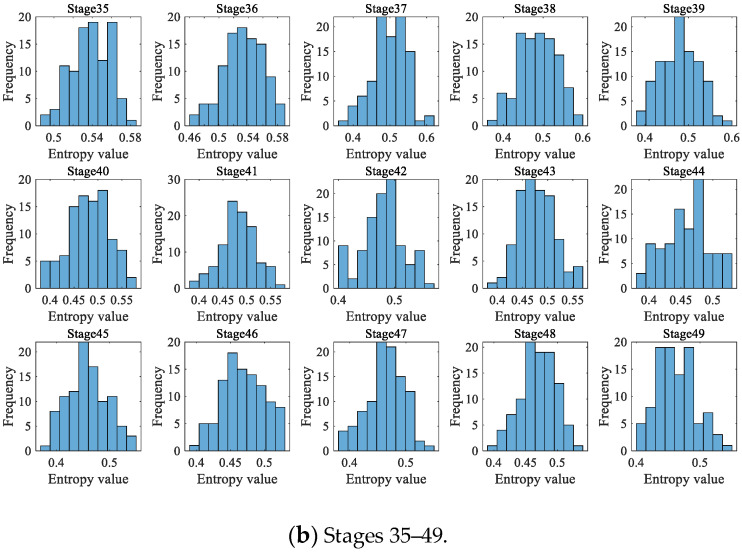
Histogram of large sample sequences of degradation stages (case 3).

As in case 1 and case 2, 200 data were taken as a degradation stage (*N* = 200), and the vibration signal was divided into 49 degradation stages. (The last degradation stage contains 240 data.) We calculated the HGE of each degradation stage to obtain the degradation feature sequence of the rolling bearings. The dynamic prediction was made for the degradation stage sequence corresponding to the last 6000 min (stage 20 to 49, a total of 30 degradation stages). The large sample sequence of 30 degradation stages predicted via the GBMC model is shown in Figure 32. We sorted the data in the large sample sequence of 30 degradation stages from small to large and then grouped them, taking Z = 10. We drew a histogram, shown in Figure 33.

**Figure 34:**
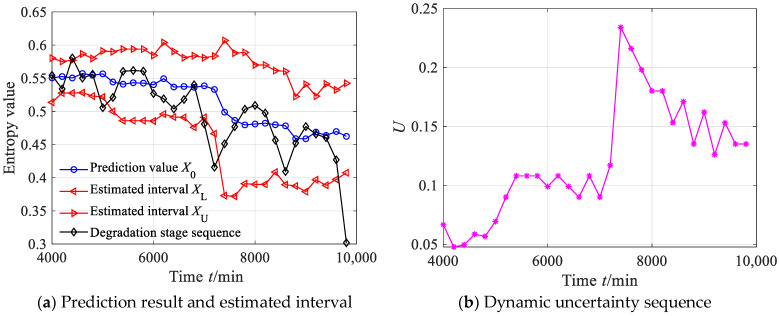
Prediction results and dynamic evaluation of rolling bearing degradation stages based on GBMC model (case 3).

According to Equations (44)–(47), the estimated true value and the estimated interval of the degradation stage sequence can be obtained, where the significance level was set as 0.05, as shown in Figure 34a. From Figure 34a, it can be seen that between 4000 and 7000 min, the predicted sequence can capture the smooth fluctuation trend of the degradation stage. Between 7000 and 10,000 min, the predicted sequence can capture the downward trend of the degradation stage. From Figure 34a, it can also be found that the estimated interval almost includes all the degradation feature values, with four degradation feature values falling outside the estimated interval. According to Equation (48), the prediction reliability *H_B_* is 83.87%, indicating that the estimated interval can effectively track the evolution trend of the degradation stage. The dynamic uncertainty of the degradation stage sequence was calculated as shown in Figure 34b. From Figure 34b, it is clear that between 4000 and 7000 min, the uncertainty stabilizes at a small-value stage, indicating that the bearing was in a stable operating period; after 7000 min, the uncertainty rapidly increased, indicating that the rolling bearing underwent serious deterioration.

**Figure 35:**
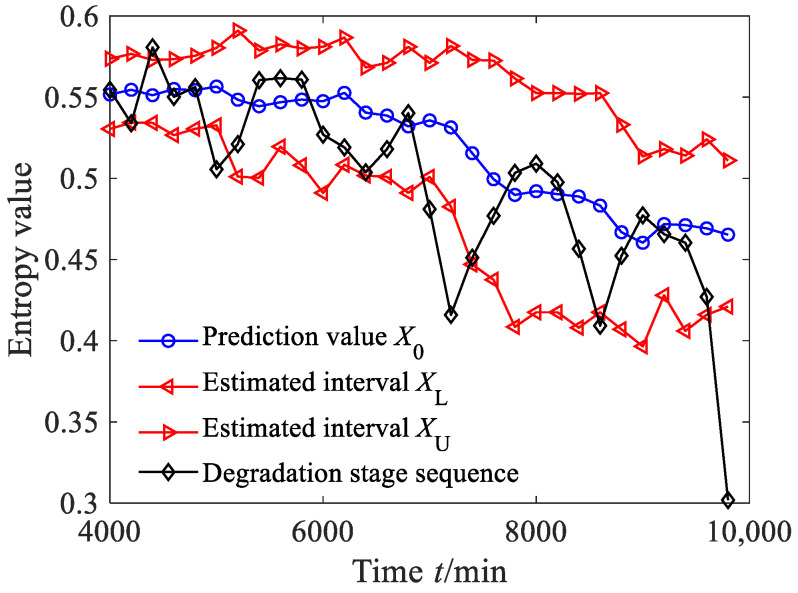
Prediction results of rolling bearing degradation stages based on GB model (case 3).

**Figure 36:**
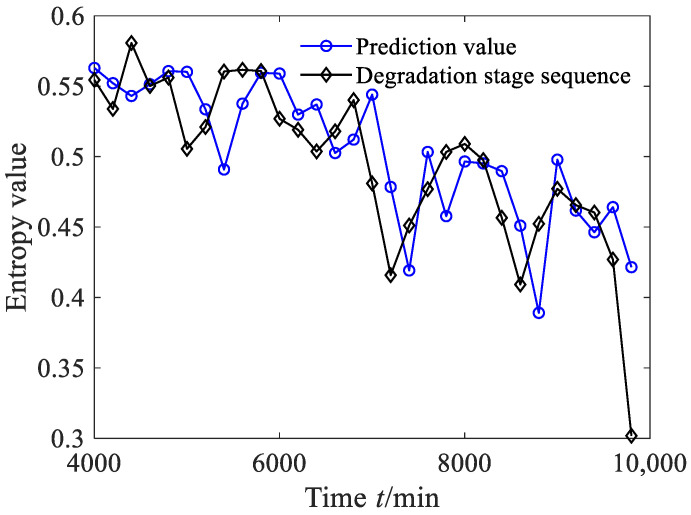
Prediction results of rolling bearing degradation stages based on AR method (case 3).

The prediction results based on the GB model are shown in Figure 35, from which it can be found that although the prediction value of the degradation stage obtained via the GB model shows an overall downward trend, reflecting to some extent the evolution trend of rolling bearing degradation stage, the prediction reliability *H_B_* is only 77.42%. The prediction results based on the AR method are shown in Figure 36, and the comparison results are listed in Table 6. Consulting Table 6, although the prediction errors and the correlation coefficient of bearing degradation stages obtained via the AR method are almost the same as that of the GBMC, the GBMC has the advantage of dynamic evaluation of prediction results.

Therefore, the above analysis indicates that the proposed GBMC-based degradation stage prediction method is superior to the GB-based and AR-based degradation stage prediction methods.

## 6. Conclusions

In this paper, a novel degradation stage prediction method based on HGE and GBMC is proposed for rolling bearings to effectively evaluate their degradation condition. Firstly, HGE is proposed to obtain multilevel degradation features at lower and higher frequencies. The superiority of HGE to HSE and HFE was verified by analyzing the simulation signals. The research indicates that compared with HFE, HSE and kurtosis, HGE can be used to extract entire information and effectively describe dynamic changes in the complex signal. Subsequently, GBMC, which combines the grey model, bootstrap method and Markov chain, is proposed to obtain more accurate degradation stage prediction results of rolling bearings compared with the GB model and AR method. Finally, three parameter indicators were employed to evaluate the prediction results from different perspectives. More significantly, the proposed novelty degradation stage prediction method was applied to three rolling bearing experimental examples. The experimental results indicate that HGE shows the best performance in extracting the degradation features of vibration signals and GBMC obtains the best degradation stage prediction results. The proposed method is promising and should be applied in feature extraction and degradation trend prediction of other mechanical equipment. In the future work, more real-world rolling bearing vibration data (such as XJTU-SY bearing datasets) should be employed to verify the effectiveness of the proposed method. A practical implication of the proposed method in future works is to achieve the online prediction of rolling bearing degradation stages. Further research can also focus on multi-source rolling bearing prognostics under variable working conditions.

## Figures and Tables

**Table 1:** The test condition of the bearing test system (case 1).

Bearing Type	Dynamic Load Rating (Cr)	P/C	Rotating Speed	Radial Load	Axial Load
7008 AC	19 KN	0.39	4000 r/min	2 KN	3.5 KN

**Table 2:** Parameters of 7008 AC bearing.

Parameter	Value
Accuracy class	P2
Inner diameter	40 mm
Outer diameter	68 mm
Width	15 mm
Dynamic load rating	18.7 KN
Static load rating	14.6 KN
Number of rolling elements	16

**Table 3:** The comparative analysis results (case 1).

	AR Method	GBMC
Average absolute error	0.0332	0.0340
Correlation coefficient	0.6094	0.7496

**Table 4:** The test condition of the bearing test system (case 2).

Bearing Type	Dynamic Load Rating (Cr)	P/C	Rotating Speed	Radial Load	Axial Load
7008 AC	19 KN	0.3	4000 r/min	4.17 KN	4.58 KN

**Table 5:** The comparative analysis results (case 2).

	AR Method	GBMC
Average absolute error	0.0503	0.0575
Correlation coefficient	0.6592	0.6593

**Table 6:** The comparative analysis results (case 3).

	AR Method	GBMC
Average absolute error	0.0310	0.0306
Correlation coefficient	0.7366	0.7007

## Data Availability

The data used to support the findings of this study are available from the corresponding author upon request.
